# Endocannabinoid Metabolome Characterization of Transitional and Mature Human Milk

**DOI:** 10.3390/nu10091294

**Published:** 2018-09-12

**Authors:** Adriana V. Gaitán, JodiAnne T. Wood, Fan Zhang, Alexandros Makriyannis, Carol J. Lammi-Keefe

**Affiliations:** 1 Louisiana State University and Louisiana State University Agricultural Center, Baton Rouge, LA 70803, USA; fzhan14@lsu.edu (F.Z.); clammi-keefe@agcenter.lsu.edu (C.J.L.-K.); 2 Center for Drug Discovery, Northeastern University, Boston, MA 02115, USA; j.wood@northeastern.edu (J.T.W.), a.makriyannis@northeastern.edu (A.M.); 3 Pennington Biomedical Research Center, Baton Rouge, LA 70803, USA

**Keywords:** fatty acids, long-chain polyunsaturated fatty acids, endocannabinoids, infant health, breast milk

## Abstract

Recognized as the gold standard, human milk (HM) is an extremely complex yet fascinating biofluid tailored to meet an infant’s nutritional requirements throughout development. Endocannabinoids and endocannabinoid-like compounds (endocannabinoid metabolome, ECM) are endogenous lipid mediators derived from long-chain polyunsaturated fatty acids that have been identified in HM. Previous research has shown that arachidonoylglycerol might play a role in establishing the infant’s suckling response during lactation by activating the type 1 cannabinoid receptor in the infant’s brain. The mechanisms of action and the role of the ECM in HM are not fully understood. Transitional and mature milk samples were collected from lactating women (*n* = 24) for ECM characterization, quantification, and to evaluate differences among the two stages. HM samples were analyzed by liquid chromatography-mass spectrometry. Identified members of the ECM were: arachidonoylethanolamine, palmitoylethanolamine, oleoylethanolamine, docosahexaenoylethanolamine, eicoapentaenoylethanolamine, eicosenoylethanolamine, arachidonoylglycerol, palmitoyglycerol, oleoylglycerol, docosahexaenoylglycerol, eicosapentaenoylglycerol, eiconenooylglycerol, arachidonic acid, docosahexaenoic acid, and eicosapentaenoic acid. Only docosahexaenoylglycerol was different across transitional and mature milk (*p* ≤ 0.05). Data from this cohort suggest that bioactive constituents in HM may also play a role in infant health and development. Future studies can be developed based on this study’s data to help elucidate specific roles for each ECM member in addition to understanding how the ECM modulates infant health.

## 1. Introduction

According to the Center for Disease Control and Prevention (2018) [1], 83.2% of infants in the United States are breastfed, with almost 60% breastfeeding at six months, almost 36% breastfeeding at 12 months, and only 24.9% meeting the global recommendation to breastfeed exclusively for six months [2,3]. The recommendation for exclusive breastfeeding during the first months following delivery is based in part on the knowledge that breast milk provides the infant with nutrients that meet his requirements during development. These beneficial nutrients include the long-chain polyunsaturated fatty acids (LCPUFAs), docosahexaenoic acid (DHA, 22:6*n*3), and arachidonic acid (ARA, 20:4*n*6), that play a role in cognitive and retinal development and growth of the infant [4]. These nutrients are transferred to the infant across the placenta during pregnancy and through breast milk after birth.

It has been shown that LCPUFAs are precursors to endocannabinoids (EC) which are endogenous lipid mediators that bind to the same receptors as *Cannabis sativa* (marijuana) [5]. Endocannabinoids have been shown to play a role in appetite and food intake [6] by activating cannabinoid receptor 1 (CB1) which is present in the central nervous system [7]. Cannabinoid receptor 1 is activated by two different EC, arachidonoylethanolamide (anandamide, AEA) and arachidonoyl glycerol (AG), both derived from *n*-6 ARA. In particular, for infant feeding behavior, AG has been demonstrated to play a role in establishing the suckling response of the neonate when nursing [8]. Evidence in mouse pups suggest that CB1 activation by AG is needed to establish the suckling response by activating the oral-motor musculature behavior needed for milk suckling [8,9,10]. Establishment of this role for AG was demonstrated after administration of a CB1 antagonist (SR141716A) to mouse pups which resulted in growth inhibition and even death by day eight after birth [8].

Recent work has indicated that EC and EC-like compounds (collectively referred to as the endocannabinoid metabolome, ECM) are present in human milk [11,12,13]. Endocannabinoid-like compounds, referred to as entourage metabolites [14], may support the activity and physiologic responses of the EC system by interacting with AEA and AG, their enzymes, or their receptors. These entourage metabolites exert cannabimimetic effects (similar pharmacological effects to those of cannabis) [15]. The ECM encompasses 15 metabolites identified to date: (i) ethanolamide derivatives: AEA, palmitoyl ethanolamide (PEA), oleoyl ethanolamide (OEA), docosahexaenoyl ethanolamide (DHEA), eicosapentaenoyl ethanolamide (EPEA), and eicosenoyl ethanolamide (EEA); (ii) glycerol derivatives: AG, palmitoyl glycerol (PG), oleoyl glycerol (OG), docosahexaenoyl glycerol (DHG), eicosapenaenoyl glycerol (EPG), eicosenoyl glycerol (EG); and (iii) precursor LCPUFAs: ARA, DHA, and eicosapentaenoic acid (EPA, 20:5*n*3). There is limited information regarding the ECM of human milk and its role in infant development. Thus, in the present study, we characterized and quantified the ECM in human milk in transitional and mature milk and evaluated if the concentrations of these metabolites changed over time. 

## 2. Materials and Methods

### 2.1. Study Design

This research project was an exploratory-longitudinal study to evaluate if there was a difference in the ECM of transitional milk (two weeks postpartum) and the ECM of mature milk (four weeks postpartum).

### 2.2. Subject Recruitment

Pregnant women from the greater Baton Rouge, Louisiana area who were planning to breastfeed for a minimum of four weeks were invited to participate in this study. Recruitment was based on intent to breastfeed. Subjects were invited to participate before delivery through private physicians’ offices and hospital prenatal clinics or by posting flyers describing the study around the community. Women who demonstrated interest in participation in the study were contacted to explain the study and for pre-screening based on the inclusion criteria: maternal age of 18–40 years at the time of delivery, full term delivery (≥37 gestational weeks), singleton birth, plan to breastfeed for at least 4 weeks, willing to provide a breast milk sample (complete breast emptying from one breast) during the morning (6–10 am), have not been breastfeeding or pregnant in the previous year. Before delivery, women were contacted again to schedule the consent process (thorough explanation of the study and for signature of the consent form). The exclusion criteria were discussed at the time of consent: any tobacco use during lactation, alcohol consumption (>1 drink per week), presumed or confirmed congenital birth defects.

Materials provided to the subjects for the study included two breast milk storage bags, instruction on how to collect the breast milk sample, and a schedule card for visits. These were provided the same day that the consent was obtained. The Louisiana State University Agricultural Center Institutional Review Board approved the study.

### 2.3. Sample Collection

Participants provided written consent and filled out a health history questionnaire that included questions about previous and current pregnancies, pregravid body mass index (BMI), and prior lactation experience. Details regarding infant birth weight and length were completed following the infant’s birth. In addition, participants provided information about education and socioeconomic status. This information was confirmed by their health care providers.

Breast milk samples were collected at two and four weeks postpartum at the participants’ homes. Participants were asked to provide a breast milk expression from a single breast (emptying a full mammary gland by collecting all the milk from that breast) [16] by using an electric breast pump. In preparation for milk collection, participants fasted for at least two hours and collections were made between 6 am and 10 am. The sample was stored under refrigeration at the participant’s house (for a maximum of 24 h) in the breast milk storage bag provided by the researcher. Samples were transported on ice to the laboratory where the milk was warmed in a 37 °C water bath, manually gently swirled to mix, and ~15 mL aliquots were made in small glass vials with Teflon-lined caps and stored at −80 °C until analyses. Information including the breast pump brand used, exclusive breastfeeding, and use of formula for supplementation were also recorded. Samples were shipped overnight on dry ice to the Center for Drug Discovery, Northeastern University, Boston, MA, USA and kept at −80 °C until analysis.

### 2.4. Sample Analysis

The breast milk samples were analyzed by liquid chromatography-mass spectrometry (LC-MS) with a state-of-the-art methodology established at the Center for Drug Discovery at Northeastern University, Boston, MA, USA. Milk samples were thawed in a 37 °C water bath and vortexed at medium speed for 10 s at room temperature. Protein precipitation was carried out with chilled acetonitrile and PBS (pH 7.4) and the addition of an internal standard mixture containing the same 15 metabolites identified followed by centrifugation (14,000× *g*, 5 min, 4 °C). The resulting supernatant was diluted with four volumes of 5% phosphoric acid followed by solid phase extraction using OASIS HLB reverse-phase chromatography cartridges (Waters Corp., Mildford, MA, USA) which were previously rinsed with methanol and water prior to loading the diluted samples. Loaded cartridges were washed with 40% aqueous methanol prior to eluting the absorbed lipids with acetonitrile. The acetonitrile fraction was evaporated to dryness under nitrogen, reconstituted in ethanol, vortexed and sonicated, and centrifuged prior to LC-MS analysis. The autosampler was kept at 4 °C to prevent analyte degradation. A TSQ Quantum Ultra triple quadrupole mass spectrometer (Thermo Electron, San Jose, CA, USA) with an Agilent 1100 liquid chromatograph (Agilent Technologies, Wilmington, DE, USA) at the front end was used for identification and quantification. Separation of analytes was carried out using an Agilent 2.1 × 50 mm, 5 μm Zorbax SB-CN column [17,18] with gradient elution using 10 mM ammonium acetate (pH 7.3) and methanol (flow rate, 0.5 mL/min). Elution of fatty acids was achieved while the mass spectrometer was in negative ionization mode, followed by a change in the mass spectrometer to positive ionization mode for elution of ethanolamine and glycerol esters. Eluted peaks were ionized via atmospheric pressure chemical ionization in multiple reaction monitoring mode as previously described [18]. Deuterated internal standards were used to derive a standard curve for each analyte and concentrations (ng/mL) of breast milk were calculated. Each sample was analyzed in triplicate and concentrations were averaged. 

### 2.5. Statistical Analyses

Statistical analyses were performed using SAS by SAS Institute, Inc., version 9.4 (Cary, NC, USA). The level of significance was set at ≤0.05. Descriptive statistics (mean, standard deviation, and range) were used for numeric variables. Repeated measures analysis of variance using proc mixed was used to assed the effect of time across the two different time points on the concentrations of members of the ECM.

## 3. Results

One hundred thirty-one potential participants were invited to participate in the study from which 31 consented to participate. Seven women dropped out during the study; thus, data from 24 participants was included in the study. Table 1 provides the participants’ characteristics. Lactating women in the study were between 18 years old and 39 years old.

Table 2 shows the constituents of the ECM at two weeks (transitional milk) and four weeks (mature milk) postpartum. Standard curves for each metabolite were linear and had regression values ≥0.99, except for PG which was 0.98. Extraction efficiencies were greater than 80%, except for OG which was greater than 78%. The main metabolite present in the fatty acids group was ARA accounting for more than 60% of that fraction. In the ethanolamide group, OEA accounted for more than 50% of that portion, and PG in the glycerol group accounted for more than 90%. Eicosenoyl ethanolamide and EPG were present in the lowest concentrations in the ethanolamide and glycerol groups, respectively.

Twenty-one percent of the samples for EPEA and 27% of the samples for EEA were below the standard curve and 21% of the samples for PG were above although values were close to the curve for the latter. Therefore, those results should be interpreted with caution. Only DHEA demonstrated a time effect (*p* ≤ 0.05) across the two different time points postpartum (transitional (two weeks) versus mature (four weeks) milk) with higher concentrations in transitional milk. Overall, breast milk glycerol group concentrations were higher than those of the ethanolamides.

Combining the two time points together to evaluate relationships, it was observed that there were significant correlations between the precursor LCPUFA and its derived EC. Results are showed in Table 3.

## 4. Discussion

Our study has characterized the ECM in transitional and mature human milk to explore differences in the ECM concentrations at these two stages of breast milk production. The mechanisms of action and the roles of the ECM in both breast milk and for infant development are not fully described/understood. Understanding the bioactive components (i.e., ECM) in breast milk contributes to the body of research that supports the importance of breast milk for infant nourishment and development.

In this exploratory study, we have characterized the ECM of transitional and mature milk. Our results showed that only DHEA, a derivative of DHA conjugated with ethanolamine, was different across the two different time points (*p* ≤ 0.05) with higher concentrations in transitional milk. Research evaluating the role of the endocannabinoid system (ECS) in infant feeding behavior has been focused on the activation of CB1 when binding to AG, which in turn activates the oral-motor musculature needed for milk suckling [8]. However, DHEA has also been shown to be an agonist to CB1 [19]. Although the role of DHEA in food intake has not been studied, it may be hypothesized that by binding to the same receptor as AG, DHEA exerts some of the same activities. In addition, as DHA plays a key role in infant cognitive development [20,21] and is a precursor to DHEA, it is plausible that DHEA also supports brain development. Moreover, the development of the hippocampus, a brain area related to learning and memory, has been shown to be supported by DHEA [22].

Scarce data are available for a comparison with our current results. However, the earliest study by Fride et al. (2001) [8] that established a role for the ECS in mouse pup suckling and growth, also analyzed milk from various sources including human milk. Even though the study by Fride et al. (2001) did not specify the number of milk samples analyzed, our results follow the same pattern in demonstrating that PG is present in human milk in higher concentrations than AG. Furthermore, a study by Di Marzo et al. (1998) [23] reported 330 ng/mL of AG in mature human milk, a concentration very similar to our result of 312.11 ng/mL, and indicated that AG is found in human milk in higher concentrations than AEA which is also demonstrated in our present results. Similarly, a study by Schuel et al. (2002) [24] in which ethanolamides were analyzed in human fluids, including mature milk, demonstrated that OEA was present in higher concentrations than PEA, and PEA in higher concentrations than AEA, as also shown in our results. In addition, our results are in line with preliminary data from our laboratory [11,12] that included the same members of the ECM that were investigated in the current study. Our results follow the same pattern in terms of the proportion of each member within each group: fatty acids, ethanolamides, and glycerols. In summary, there are only a few studies available for a comparison to the findings of our current study that support the presence of EC and EC-like metabolites in human milk.

Correlations between the precursor LCPUFAs and their ethanolamide- and glycerol-derivatives showed a more robust correlation for the precursor LCPUFA and its derived glycerol metabolites. This strong correlation between the precursor LCPUFA with its glycerol- but not its ethanolamide-derivatives, may support a more important role for the glycerols (AG, PG, OG, DHG, EPG, and EG) in establishing the suckling response of the newborn by modulating motor development and behavior. In addition, the presence of entourage metabolites (PEA, OEA, DHEA, EPEA, EEA, PG, OG, DHG, EPG, and EG), which exhibit cannabimimetic responses [14], may enhance the activity of the two most thoroughly studied EC, AG, and AEA. For example, PG has been shown to increase AG affinity to CB2 by acting as a lipid signaling mediator [18]; and PEA and OEA reduce enzymatic breakdown, cellular uptake, and degradation of AEA. These entourage metabolites may interfere with enzymatic activity as they can also act as substrates for catabolic and anabolic enzymes. In addition, the presence of these lipid mediators may prevent EC activation or deactivation. All of these interactions may also explain our finding that *n*-3 LCPUFA derivatives, both ethanolamides and glycerols, correlated with each other (DHEA-DHG (*r* = 0.61, *p* ≤ 0.01) and EPEA-EPG (*r* = 0.84, *p* ≤ 0.01)). The associations among the *n*-3 LCPUFA derivatives, but not for the *n*-6 derivatives (AEA and AG), leads to the speculation that they support the role of DHA in infant cognitive development, although their roles have not yet been fully elucidated.

To date, the mechanisms of action regarding how the ECM as a whole interacts with the ECS and its role in infant feeding behavior, and therefore infant development and growth, are still poorly understood. Our results provide evidence that there are metabolites similar to the previously described EC [5], i.e., AEA and AG, present in human milk. With an understanding of the role of the ECM and its interactions with the ECS in human milk and the infant’s brain, potential interventions could be developed for infants with difficulties latching on and for preterm infants who could be aided by the countless benefits of breast milk to ensure continued development outside the womb. While this is out of the scope of this study, it merits further exploration.

This study was limited by its small sample size (*n* = 24). Having a relatively small group of participants did not allow for further explorations between the concentrations for some of the ECM members and demographic data such as BMI and race, for example. However, this study provides an opportunity to develop hypotheses for future studies to evaluate how the ECM of breast milk may be modulated on the basis of maternal and/or infant factors.

## 5. Conclusions

Our study provides evidence that EC and EC-like metabolites are present in human milk. The findings in this study not only support the role of AG in establishing the suckling response of the newborn by activating oral-motor musculature needed for milk suckling, but also suggest that other bioactive constituents in breast milk may also play a role in infant health and development. In addition, knowing that EC-like metabolites are present in breast milk, future studies can be developed to elucidate specific roles for each member of the ECM.

## Figures and Tables

**Table 1:** Maternal-Infant Characteristics (*n* = 24).

Characteristic	Mean ± SD or % (Frequency)	
**Maternal Characteristics**		
Age (year)	30.5 ± 5.0	
Pre-pregnancy BMI (kg/m^2^)	28.0 ± 5.8	
Race		
White	71 (17)	
Black	17 (4)	
Hispanic	8 (2)	
Asian	4 (1)	
Gestational age at delivery (weeks)	39.2 ± 1.3	
Previous breastfeeding experience		
No	71 (17)	
Education		
Some high school	4 (1)	
High school	4 (1)	
Some college	21 (5)	
4-year post-high school	25 (6)	
Post-graduate	46 (11)	
Marital Status		
Married	79 (19)	
WIC participation		
No	88 (21)	
**Infant characteristics**		
Sex		
Girls	33 (8)	
Mode of delivery		
Vaginal	75 (18)	
Birth weight (lbs)	7.4 ± 0.8	
Feeding type	2 weeks	4 weeks
Exclusively breastfed	83 (20)	67 (16)

BMI, body mass index; WIC, Woman, Infant, and Children Special Supplemental Nutrition Program.

**Table 2:** Endocannabinoid Metabolome of Human Milk.

Metabolite	Transitional Milk ^1^	Mature Milk ^1^	*p* Value ^2^
Fatty Acids			
ARA	2818.96 ± 580.77	7030.33 ± 3638.67	0.2451
DHA	2031.17 ± 486.39	2384.71 ± 1140.13	0.7569
EPA	381.49 ± 131.91	1362.93 ± 933.24	0.2979
Ethanolamides			
AEA	0.15 ± 0.05	0.08 ± 0.01	0.1772
PEA	0.90 ± 0.10	0.74 ± 0.08	0.1095
OEA	1.48 ± 0.24	1.12 ± 0.10	0.0841
DHEA	0.11 ± 0.01	0.07 ± 0.01	**0.0022**
EPEA ^3^	0.07 ± 0.03	0.11 ± 0.04	0.5184
EEA ^3^	0.03 ± 0.01	0.03 ± 0.00	0.2382
Glycerol esters			
AG	166.85 ± 36.30	312.11 ± 119.97	0.2550
PG ^4^	37,477.67 ± 7296.61	110,091.70 ± 54,443.90	0.1905
OG	4059.33 ± 716.85	7719.96 ± 2269.68	0.1225
DHG	673.50 ± 198.01	866.30 ± 383.60	0.6352
EPG	24.70 ± 7.70	61.99 ± 28.85	0.2161
EG	242.48 ± 66.75	899.37 ± 509.86	0.2078

All data are presented in ng/mL and are mean ± SE. Significant difference marked in bold. ^1^ Two and four weeks postpartum. ^2^ *p* value represents the effect of time across the two time points. ^3^ Some values were below the standard curve. ^4^ Some values were above the standard curve. ARA, arachidonic acid; DHA, docosahexaenoic acid; EPA, eicosapentaenoic acid; AEA, Anandamide; PEA, palmitoyl ethanolamide; OEA, oleoyl ethanolamide; DHEA, docosahexaenoyl ethanolamide; EPEA, eicosapentaenoyl ethanolamide; EEA, eicosenoyl ethanolamide; AG, arachidonoyl glycerol; PG, palmitoyl glycerol; OG, oleoyl glycerol; DHG, docosahexaenoyl glycerol; EPG, eicosapenaenoyl glycerol; EG, eicosenoyl glycerol.

**Table 3:** Correlations between the Parent Fatty Acid and its Derived Metabolites.

Fatty Acid	Metabolite	Pearson Correlation Coefficient ^1^
ARA	AG	0.88
DHA	DHEA	0.69
DHA	DHG	0.95
EPA	EPEA	0.80
EPA	EPG	0.91

^1^*p* ≤ 0.01. ARA, arachidonic acid; DHA, docosahexaenoic acid; EPA, eicosapentaenoic acid; AG, arachidonoyl glycerol; DHEA, docosahexaenoyl ethanolamide; DHG, docosahexaenoyl glycerol; EPEA, eicosapentaenoyl ethanolamide; EPG, eicosapenaenoyl glycerol.
